# Supplementary material for: Parallel evolution of influenza across multiple spatiotemporal scales
Source: eLife. 2017 Jun 27;6:e26875. doi: 10.7554/eLife.26875 (PMC5487208; doi:10.7554/eLife.26875)
Supplement: Figure 2—source data 1. — 5’ primer tail sequence is indicated in plain text, homology to the U12/U13 regions in bold text, and gene-specific sequence in bold underlined text. Primers were modified from the universal primers described in (Hoffmann et al., 2001) to account for the A/G polymorphism at the U4 site of the U12 region. DOI: http://dx.doi.org/10.7554/eLife.26875.006 [file elife-26875-fig2-data1.docx]

**Figure 2—Source Data 1. Primers used for viral deep sequencing.** 5’ primer tail sequence is indicated in plain text, homology to the U12/U13 regions in **bold text**, and gene-specific sequence in **bold underlined text**. Primers were modified from the universal primers described in Hoffmann 2001(Hoffmann et al. 2001) to account for the A/G polymorphism at the U4 site of the U12 region.

| reaction | primer name | sequence | ratio |
| --- | --- | --- | --- |
| reverse transcription | 5’-Hoffmann-U12-A4 | TATTGGTCTCAGGG**AGCAAAAGCAGG** | 1 |
|  | 5’-Hoffmann-U12-G4 | TATTGGTCTCAGGG**AGCGAAAGCAGG** | 1 |
| PCR | 5’-Hoffmann-Ba-PB2-1-A4 | TATTGGTCTCAGGG**AGCAAAAGCAGGTC** | 1 |
|  | 5’-Hoffmann-Ba-PB2-1-G4 | TATTGGTCTCAGGG**AGCGAAAGCAGGTC** | 1 |
|  | 3’-Hoffmann-Ba-PB2-2341R | ATATGGTCTCGTATT**AGTAGAAACAAGGTCGTTT** | 2 |
|  | 5’-Hoffmann-Bm-PB1-1-A4 | TATTGGTCTCAGGG**AGCAAAAGCAGGCA** | 1 |
|  | 5’-Hoffmann-Bm-PB1-1-G4 | TATTGGTCTCAGGG**AGCGAAAGCAGGCA** | 1 |
|  | 3’-Hoffmann-Bm-PB1-2341R | ATATGGTCTCGTATT**AGTAGAAACAAGGCATTT** | 2 |
|  | 5’-Hoffmann-Bm-PA-1-A4 | TATTGGTCTCAGGG**AGCAAAAGCAGGTAC** | 1 |
|  | 5’-Hoffmann-Bm-PA-1-G4 | TATTGGTCTCAGGG**AGCGAAAGCAGGTAC** | 1 |
|  | 3’-Hoffmann-Bm-PA-2233R | ATATGGTCTCGTATT**AGTAGAAACAAGGTACTT** | 2 |
|  | 5’-Hoffmann-Bm-HA-1-A4 | TATTGGTCTCAGGG**AGCAAAAGCAGGGG** | 1 |
|  | 5’-Hoffmann-Bm-HA-1-G4 | TATTGGTCTCAGGG**AGCGAAAGCAGGGG** | 1 |
|  | 3’-Hoffmann-Bm-HA-890R | ATATGGTCTCGTATT**AGTAGAAACAAGGGTGTTTT** | 2 |
|  | 5’-Hoffmann-Bm-NP-1-A4 | TATTGGTCTCAGGG**AGCAAAAGCAGGGTA** | 1 |
|  | 5’-Hoffmann-Bm-NP-1-G4 | TATTGGTCTCAGGG**AGCGAAAGCAGGGTA** | 1 |
|  | 3’-Hoffmann-Bm-NP-1565R | ATATGGTCTCGTATT**AGTAGAAACAAGGGTATTTTT** | 2 |
|  | 5’-Hoffmann-Ba-NA-1-A4 | TATTGGTCTCAGGG**AGCAAAAGCAGGAGT** | 1 |
|  | 5’-Hoffmann-Ba-NA-1-G4 | TATTGGTCTCAGGG**AGCGAAAGCAGGAGT** | 1 |
|  | 3’-Hoffmann-Ba-NA-1413R | ATATGGTCTCGTATT**AGTAGAAACAAGGAGTTTTTT** | 2 |
|  | 5’-Hoffmann-Bm-M-1-A4 | TATTGGTCTCAGGG**AGCAAAAGCAGGTAG** | 1 |
|  | 5’-Hoffmann-Bm-M-1-G4 | TATTGGTCTCAGGG**AGCGAAAGCAGGTAG** | 1 |
|  | 3’-Hoffmann-Bm-M-1027R | ATATGGTCTCGTATT**AGTAGAAACAAGGTAGTTTTT** | 2 |
|  | 5’-Hoffmann-Bm-NS-1-A4 | TATTGGTCTCAGGG**AGCAAAAGCAGGGTG** | 1 |
|  | 5’-Hoffmann-Bm-NS-1-G4 | TATTGGTCTCAGGG**AGCGAAAGCAGGGTG** | 1 |
|  | 3’-Hoffmann-Bm-HA-890R | see above | 2 |
