# Supplementary material for: Parallel evolution of influenza across multiple spatiotemporal scales
Source: eLife. 2017 Jun 27;6:e26875. doi: 10.7554/eLife.26875 (PMC5487208; doi:10.7554/eLife.26875)
Supplement: Figure 4—source data 1. — DOI: http://dx.doi.org/10.7554/eLife.26875.019 [file elife-26875-fig4-data1.docx]

**Figure 4—Source Data 1. Overlap of mutations at the within-host and global scales.**

| **Gene** | **Within-host variants** | **Parallel within-host variants** | **Global variants** | **Overlap,**  **within-host and global variants** | **Overlap, parallel within-host and global variants** |
| --- | --- | --- | --- | --- | --- |
| Hemagglutinin | 19 | 5 | 79 | 8 | 4 |
| Neuraminidase | 17 | 3 | 67 | 3 | 1 |
| Other genes | 47 | 2 | 176 | 5 | 0 |
